# Current challenges for global equity related to the implementation of artificial intelligence in pediatric imaging

**DOI:** 10.1007/s00247-026-06717-9

**Published:** 2026-07-23

**Authors:** Rutger A. J. Nievelstein, Amit Gupta, Joanna Kasznia-Brown, Nasreen Mahomed, Jaishree Naidoo, Marla B. K. Sammer

**Affiliations:** 1 Department of Pediatric Radiology & Nuclear Medicine, University Medical Center Utrecht/Wilhelmina Children’s Hospital, P.O. Box 85500, 3508 GA Utrecht, Netherlands; 2 Department of Diagnostic and Interventional Oncoradiology, All India Institute of Medical Sciences, New Delhi, India; 3 Department of Pediatric Radiology, Musgrove Park Hospital, University of Bristol, Bristol, UK; 4 Department of Radiology, Faculty of Health Sciences, University of the Witwatersrand, Johannesburg, South Africa; 5 Paediatric Diagnostic Imaging, Dr. J. Naidoo Inc., Johannesburg, South Africa; 6 Envisionit Deep AI Ltd, Great Ormond Street Hospital, Cobham, UK; 7 Edward B. Singleton Department of Radiology, Texas Children’s Hospital, Baylor College of Medicine, Houston, USA

**Keywords:** Artificial intelligence, Child health, Equity, Global, Imaging, Pediatrics, Radiology

## Abstract

Artificial intelligence (AI) is rapidly transforming medical imaging, offering unprecedented opportunities to enhance diagnostic accuracy, streamline workflows, and personalize care. However, its integration into pediatric radiology presents unique challenges that threaten to widen existing global health disparities if not addressed thoughtfully. These challenges are related to data inequality and bias in model development, infrastructure disparities, regulatory and ethical gaps, workforce capacity and training gaps, language and localization barriers, costs and commercialization, and sustainability and long-term support issues. This article, written by representatives from the World Federation of Pediatric Imaging (WFPI), will address the key barriers to global validation and implementation of AI in pediatric radiology and how they can be addressed. By linking these domains to practical actions and responsibilities and outlining a time-sequenced roadmap, this paper provides an equity-focused, pediatric-specific framework to guide global implementation.

**Graphical Abstract:**

**Figure:**
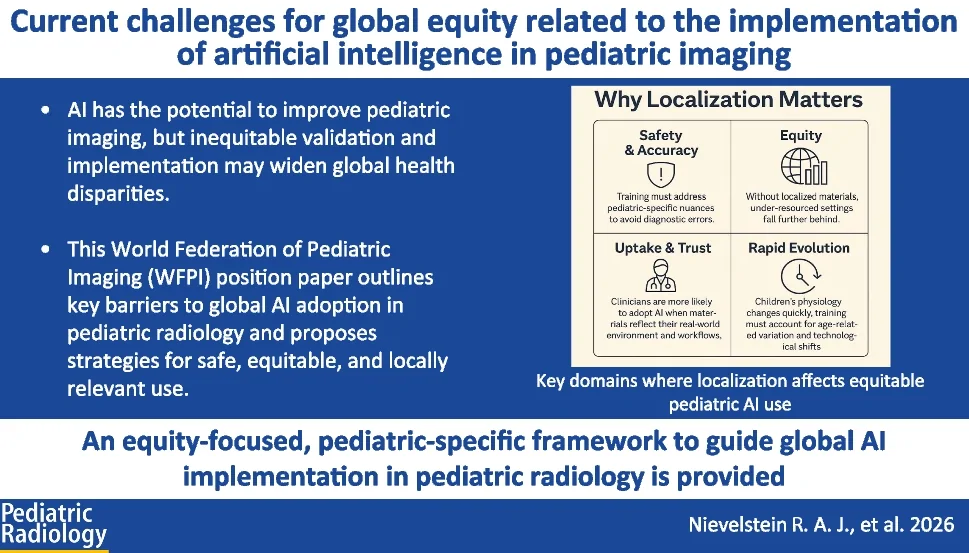

## Introduction

Artificial intelligence (AI) is transforming healthcare by offering tools that can enhance diagnostic accuracy, reduce clinician workload, and improve patient outcomes. Particularly in the field of radiology, AI innovation and integration have become leading topics of research and development [1]. However, most AI tools validated and approved for clinical use are designed for adult populations, with only a small fraction explicitly validated for children [2]. This reflects broader systemic inequities, including limited high-quality pediatric imaging data, limited funding, smaller market size, and stricter legal and ethical constraints related to pediatric data collection. Moreover, the benefits of AI innovations are not equitably distributed across the globe.

The majority of AI research and development in pediatric radiology is concentrated in high-income countries (HIC), whereas it is estimated that approximately 90% of the global child and adolescent population resides in low- and middle-income countries (LMIC) [3, 4]. Despite this demographic reality, only a small fraction of AI studies and tools are designed with the LMIC context in mind, while AI tools trained on data from HIC may not generalize well to LMIC populations due to differences in disease prevalence, imaging protocols, and patient demographics. In addition, unique challenges for the development of AI tools in pediatric radiology include age-related physiological and anatomical differences, and variations in imaging technique related to size and cooperation of the child, as well as radiation protection considerations.

In this context, it is important to distinguish equality from equity, as the two concepts imply fundamentally different implementation strategies. *Equality* refers to providing the same AI tool to every healthcare setting or population. Although this approach seems fair, it assumes that all hospitals, communities, and/or patient groups start from the same baseline condition, which rarely holds true in pediatric radiology. On the other hand, *equity* focuses on developing and implementing AI to achieve comparable outcomes across diverse populations, recognizing that different settings face different barriers and therefore require tailored solutions. In other words, *equality* standardizes inputs, and *equity* standardizes outcomes. This distinction is essential as equality may amplify existing disparities, whereas an equity-driven design and deployment of AI tools can improve diagnostic quality and access for all children, independent of the geographic and socioeconomic setting.

Therefore, to ensure that AI contributes meaningfully to global pediatric radiology and child health, there is a pressing need for international collaboration to support inclusive data sharing, capacity building, and investment in LMIC-specific AI research and infrastructure. This position paper, written by representatives from the World Federation of Pediatric Imaging (WFPI), discusses the key barriers related to the global validation and implementation of AI in pediatric radiology and how they can be addressed.

## Approach

The content of this position paper is based on the authors’ own experiences as well as a consensus discussion among the co-authors, supplemented by a targeted search of the scientific literature, online guideline documents, and websites of relevant global health organizations. It explicitly does not include a systematic review of scientific literature with evidence quality assessment.

To link system-level barriers to equity-related outcomes in pediatric imaging, the paper is structured along the following seven domains: data inequality and bias in model development, infrastructure disparities, regulatory and ethical gaps, workforce capacity and training gaps, language and localization barriers, costs and commercialization, and sustainability and long-term support. These domains are used to describe barriers and solutions across the AI lifecycle, from design and development to deployment and maintenance. An overview of the main AI model families relevant to pediatric imaging, their typical applications, and their relationships to these equity domains is provided in Table 1.

**Table 1:** Artificial intelligence (AI) model families and their relation to equity challenges in pediatric radiology

Model family or framework	Typical pediatric applications	Key equity dependencies and risks	Equity-focused strategies and requirements	Linked equity domains^a^
Classical machine learning and radiomics	Bone age, quantitative ultrasound or CT features-based classification	Protocol sensitivity, narrow feature definitions, limited generalizability	Standardize inputs, transparent features, subgroup metrics, external validation	1, 4, 6, 7
Two-dimensional CNN classification and detection	Chest radiograph triage, fractures, lines and tubes	Needs large diverse labelled datasets, age and device shift	Balance age bands, multisite data, stratified reporting, edge-friendly deployment	1, 2, 4, 6, 7
Three-dimensional and segmentation networks	Brain tumor, organ and lesion segmentation, volumetry	High compute, heavy annotation burden, workflow dependence	Phased rollout, shared annotation standards, label-efficient training, drift monitoring	1, 2, 4, 6, 7
Vision transformers and pretrained vision models	Transfer learning for small datasets, multi-task imaging	Pretraining bias, compute-intensive tuning, limited transparency	Local validation, efficient tuning, monitoring	1, 2, 4, 6, 7
Generative image models and reconstruction	Image reconstruction and quality improvement, denoising, faster MRI	Bias amplification, privacy leakage risk	Restrict use cases, privacy review, validate subgroup gains	1, 3, 6, 7
Large language models and NLP tools	Report structuring, translation, patient education, decision support	Low resource language gaps, hallucinations, terminology drift	Local lexicons, human oversight for safety steps, error tracking, guardrails	3, 4, 5, 6, 7
Federated learning and privacy preserving training	Multisite training without data pooling, rare disease models	Governance and label alignment needs, uneven site contribution	Harmonize labels, support low resource sites, site-level reporting, shared oversight	1, 2, 3, 4, 7

*AI*, artificial intelligence, *CNN*, convolutional neural network, *CT*, computed tomography, *MRI* magnetic resonance imaging, *NLP*, natural language processing

^a^Domain numbers refer to the seven equity domains discussed in this manuscript: (1) data inequality and bias in model development, (2) infrastructure disparities, (3) regulatory and ethical gaps, (4) workforce capacity and training gaps, (5) language and localization barriers, (6) costs and commercialization, (7) sustainability and long-term support

## Equity domains

### Data inequality and bias in model development

AI models for pediatric imaging are overwhelmingly built on data from HIC, leading to significant *geographic data inequalities*. A 2021 systematic review found that 82% of machine learning studies in child health came from HIC, while only 3% came from LMIC [5]. Moreover, children are grossly underrepresented in medical imaging datasets: one recent analysis of 181 public imaging datasets found that children made up <1% of all patients in these datasets [6]. In many cases, pediatric images are simply not collected, shared, or labeled at scale due to limited research funding, stricter consent requirements, and privacy concerns, resulting in pediatric datasets that are small, less diverse, and concentrated in tertiary hospitals in North America or Europe [5, 7].

Bias in AI model development is also related to age and normal development. Many AI systems treat pediatrics as one category despite major physiological and anatomical differences across infants, children, and adolescents, a problem described as *age-related algorithmic bias* [8]. Normal variations, including bone density or growth plate appearances across ethnicities, can be misclassified as pathology when absent from training data. These limitations can produce systematic errors and higher misdiagnosis rates in underrepresented groups, and thus, widen existing disparities [7]. A real-world example is the poor performance of an adult-trained chest radiograph model for cardiomegaly in infants, with error rates approaching 50% due to differences in infant chest anatomy [6]. Underrepresentation of specific age bands within pediatric populations, such as neonates and young adolescents, can lead to uneven performance highlighting the need for balanced data across all age subgroups.

*Differences in disease prevalence* further amplify this problem. Conditions such as pediatric tuberculosis and malnutrition are more prevalent in LMICs, yet models trained mainly on HIC data often have limited exposure to such cases during development and, therefore, may fail to recognize them in practice [9].

#### Solutions and enablers

Unless developers account for these geographic, demographic, and age-related diversity, AI will continue to underperform for the very populations that stand to benefit the most [7]. Therefore, to ensure fairness, rigorous external validation of pediatric AI tools in different geographic and ethnic groups before deployment is strongly recommended. Developers are also encouraged to mandate performance metrics stratified by pediatric subgroups, including age, sex, ethnicity, and geography, to better identify disparities and guide retraining, as recommended by reporting guidelines like CONSORT-AI and the pediatric-specific ACCEPT-AI framework [8].

In addition to external validation, AI tools should undergo formal benchmarking against the target pediatric population prior to implementation. Local benchmarking enables assessment of discrimination, calibration, and subgroup performance under real-world conditions, and identifies the need for threshold adjustment, recalibration, or retraining before clinical use. This step is essential to ensure safety, reliability, and equity across diverse pediatric populations.

On the technical front, researchers are exploring solutions like federated learning to mitigate data disparities. Federated learning allows multiple hospitals to collaboratively train an AI model without pooling their raw data, thus respecting privacy while still benefiting from diverse data [10]. In a recent international study of pediatric brain tumors, a federated approach across 19 sites on multiple continents achieved nearly the same accuracy as centralized training and importantly improved the model’s generalization on out-of-network data by 20–30% [11].

Several professional bodies have called for inclusive pediatric AI development. The American College of Radiology’s Pediatric AI workgroup, for instance, highlights the need for pediatric-specific AI solutions and has launched the “Image IntelliGently” campaign to promote safe, effective AI use for children [12]. The European Society of Paediatric Radiology (ESPR) AI taskforce and the World Federation of Pediatric Imaging (WFPI) advocate for increased data sharing and collaboration across borders [13]. The Africa-Canada AI and Data Innovation Consortium links institutions in HIC and African countries to jointly develop health AI solutions [8]. Such partnerships facilitate the curation of diverse pediatric imaging databases, including diseases and populations that are otherwise absent from Western-centric datasets. To support such efforts, greater transparency is required in reporting dataset composition, including age ranges, geographic origin, and ethnic representation, alongside clearer vendor documentation of reference standards, biases, and limitations.

### Infrastructure disparities

AI-driven pediatric imaging depends on *key infrastructure* such as reliable power, digital imaging equipment, picture archiving and communication system (PACS), broadband connectivity, and adequate computational resources. Many facilities in LMICs still operate with unreliable electricity or none at all, which constrains any digital imaging or AI workflow [14]. Scarce radiology resources (including modern equipment and IT infrastructure) directly impede the adoption of AI in LMIC institutions. In some low-income settings, even conventional X-ray units remain analog, and internet bandwidth is insufficient for transmitting large imaging files, effectively restricting most AI solutions to well-resourced centers [15, 16]. Likewise, the absence of electronic medical records and PACS in many facilities means critical data and images cannot be easily integrated into AI workflows. Indeed, the underrepresentation of LMIC pediatric data in AI training is partly attributable to fragmented digitization and weak IT infrastructure, reinforcing biases in AI tool performance [8, 17].

#### Solutions and enablers

Closing this gap requires staged, context-aware infrastructure building. Power resilience and basic power conditioning can enable imaging, servers, and networking to function consistently [14]. Connectivity solutions must match local realities. Hybrid online and offline workflows with local caching and store-and-forward can facilitate sustained AI use when bandwidth is weak. Portable digital radiography paired with offline AI for tuberculosis screening illustrates the feasibility of on-device analysis with later synchronization in remote programs [18]. Where cloud access is unreliable, an edge-first approach using compressed models reduces computing needs, energy use, and latency while preserving accuracy [19]. Interoperability reduces cost and complexity by using standard-based technology stacks. These include DICOMweb for accessing medical images in DICOM format and HL7 FHIR for clinical data integration, along with open, lightweight tools such as Orthanc and the OHIF DICOM viewer [20]. National alignment with digital health architectures and maturity roadmaps can help sequence investments, aggregate demand, and sustain scale. Recent guidance from the World Bank and the World Health Organization (WHO) Global Initiative on Digital Health provides practical frameworks for country-level implementation [21, 22]. Operational readiness assessments, such as RAD-AID Radiology-Readiness, can identify power, workforce, networking, and maintenance gaps before deployment and guide phased rollout in LMIC hospitals [15, 23]. Finally, continuous monitoring including post-deployment tracking of uptime, latency, and equity metrics, exemplified by the ACR Assess-AI initiative, supports governance and course correction over time [24].

### Regulatory and ethical gaps

The integration of AI into pediatric imaging raises several *ethical*, *legal*, and* moral considerations* [25–29]. Children represent a vulnerable patient population, with unique challenges and considerations of autonomy, safety, consent, and justice, which require particular attention. To support equitable collaboration, clear governance for data ethics and sovereignty is needed, including agreed standards for how pediatric imaging data are deidentified, shared, accessed, and used. In parallel, safeguards to prevent exploitation and digital colonialism of imaging data from LMIC for the development and production of expensive AI tools that preferentially benefit HIC are also required [30–32]. In line with this, RAD-AID developed the RAD-AID Friendship Data Trust, which offers healthcare institutions from LMICs the opportunity to share anonymized data to a not-for-profit collective data trust [33]. In this way, RAD-AID aims to engage AI developers that are willing to develop and make AI software available at no cost to these institutions.

For children, *informed consent* is particularly complex because minors cannot provide legal consent, and parental consent may not always adequately reflect long-term considerations regarding data use, storage, or secondary research applications. Therefore, transparent ethical codes are essential to respect the principles of privacy, proportionality, and the child’s evolving rights over their data [2, 15].

Another hurdle is the *cloud versus on-premises use* [33]. While many AI tools use cloud architecture for processing, restrictive or unclear regulations on the use of cloud for health data form a major barrier to the adoption of AI in LMICs. On-premises use may seem an alternative, but it can be less secure and requires ongoing updates and on-site technical support. Therefore, regulatory frameworks are needed that define when cloud storage and processing are permitted, how cross-border data flows are governed, and what minimum security and audit requirements apply. These frameworks should also include data management rules for how imaging data are stored, deidentified, accessed, transferred, and processed, supported by clear data sharing agreements. Reducing legal uncertainty is important to avoid delaying investments needed in LMIC.

The clinical use of AI also raises critical questions on *accountability and explainability*. If an AI system contributes to diagnostic errors through bias, dataset shift, or technical failure, responsibility may be unclear across the radiologist, vendor, and institution. In pediatrics, where consequences of delayed or missed diagnoses can be lifelong, the threshold for acceptable error is particularly low. Consequently, ethical implementation requires clear lines of accountability, including governance systems for validation, monitoring for drift, and regulatory mechanisms mandating transparency of algorithm design and intended use. Clinicians must be able to understand and evaluate AI outputs and exercise critical judgement, rather than relying on automated output without oversight.

The introduction of AI may also alter the *clinician–patient relationship* [34]. Trust is central in pediatric care, where parents and guardians rely on (pediatric) radiologists to interpret complex findings and advocate for the child’s best interests. If families believe that critical decisions are delegated to algorithms, the relational foundation of care may be undermined. Ethical practice demands that AI be used to augment, not replace, the human dimensions of care such as empathy, contextual judgement, and advocacy for the child’s welfare.

#### Solutions and enablers

Practical enablers for ethical and equitable implementation include clear governance for data ethics and sovereignty, consent processes that protect children’s rights, and regulatory clarity for cloud and on-premises deployment in different jurisdictions. Additional priorities are defined accountability across clinicians, institutions, and vendors, mandated expectations for transparency and explainability, and routine monitoring for drift and safety events. Embedding these requirements into procurement, training, and international collaborations can support an ethical use of AI in pediatric imaging across diverse settings.

### Workforce capacity and training gaps

**Fig. 1:**
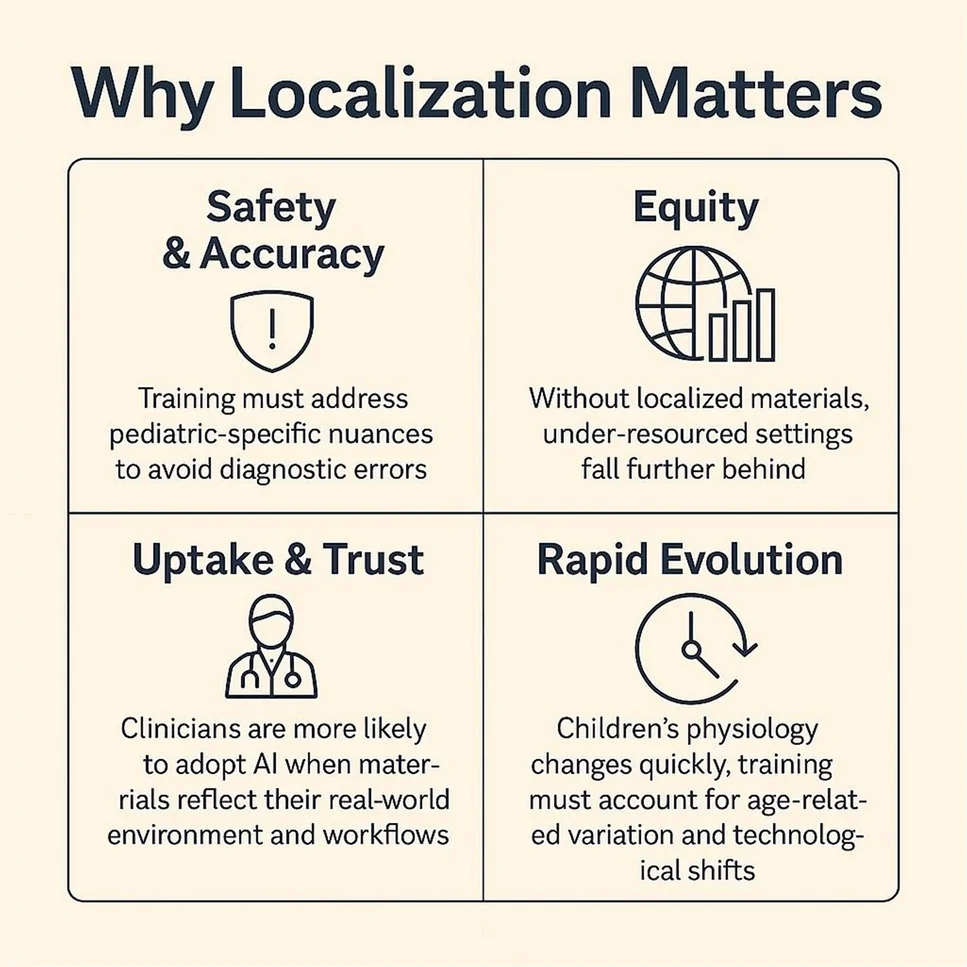
Key domains where localization directly impacts safe and equitable pediatric AI use. Tailoring training and curricula ensures diagnostic accuracy, equitable access across resource settings, clinician trust through context-specific materials, and adaptation to the rapid physiological and technological changes unique to pediatrics

Deployment of AI in pediatric radiology is constrained by critical gaps in *workforce capacity* and training. In Fig. 1, a practical checklist is given for planning implementation, linking localization of training and workflows to safety, access, trust, and pediatric-specifc adaptation (Fig. 1). A successful implementation requires pediatric radiologists and support from data scientists, IT specialists, and technicians trained in AI, which are often limited in LMICs. A global shortage of pediatric radiologists has been well documented, particularly in LMIC, where access to the expert opinion is limited and often concentrated in tertiary centers [25, 35]. The implementation of AI places additional demands on this already limited workforce, including tasks such as dataset curation, bias evaluation, performance monitoring, and post-deployment assessments of algorithms, without relevant support and training [30]. Yet only a few departments are appointing clinical AI leads with protected time to guide procurement, governance, and clinical integration [36].

The absence of in-house data engineers and informatics support leaves many institutions dependent on external vendors to provide end-to-end solutions, which may limit local autonomy, reduce transparency, create dependency, and increase costs. Smaller centers, especially in LMICs, often lack the resources and personnel to critically evaluate vendor claims or to negotiate for tools which are specific and validated in pediatric populations. Unless these structural gaps are addressed, the adoption of AI may risk widening rather than narrowing global disparities in access to safe imaging services.

Current radiology training curricula, including for pediatric subspecialties, provide little *structured education on AI* [13]. Most programs lack formal modules and often remain focused on broad concepts of AI rather than practical competencies such as dataset annotation, validation methodology, or the supervision of algorithm outputs in clinical practice. Moreover, there is limited structured teaching on the pediatric-specific challenges, including small, heterogeneous datasets, rare conditions, and safety implications across growth and development [12, 13].

Crucially, this training gap extends beyond radiologists. Radiographers, physicists, and allied health professionals have a crucial role in scan acquisition, protocol optimization, and quality control, but are rarely included in AI-focused education. Likewise, a few opportunities exist for inter-professional training or cross-disciplinary collaboration with computer scientists and engineers, despite being essential for safe implementation. Without these skills, healthcare professionals remain ill-prepared to supervise AI algorithms, critically appraise their outputs, or respond effectively when AI-generated results conflict with clinical evidence [12, 36].

#### Solutions and enablers

Without investment in workforce development and structured training, AI could unintentionally deepen the disparities in the global healthcare. Institutions in HIC with dedicated AI leadership and technical support are positioned to advance rapidly, while centers in LMIC may struggle to integrate tools safely and effectively. Therefore, protected roles for clinical AI leadership, coupled with investment in multidisciplinary training that includes (pediatric) radiologists, radiographers, physicists, and IT specialists, are crucial for ensuring that AI in pediatric imaging evolves in a manner that is safe, equitable, sustainable, and globally relevant. This includes embedding AI competencies into pediatric radiology curricula, developing accessible online and modular training platforms, and creating international partnerships to strengthen capacity across all regions.

### Language and localization barriers

**Fig. 2:**
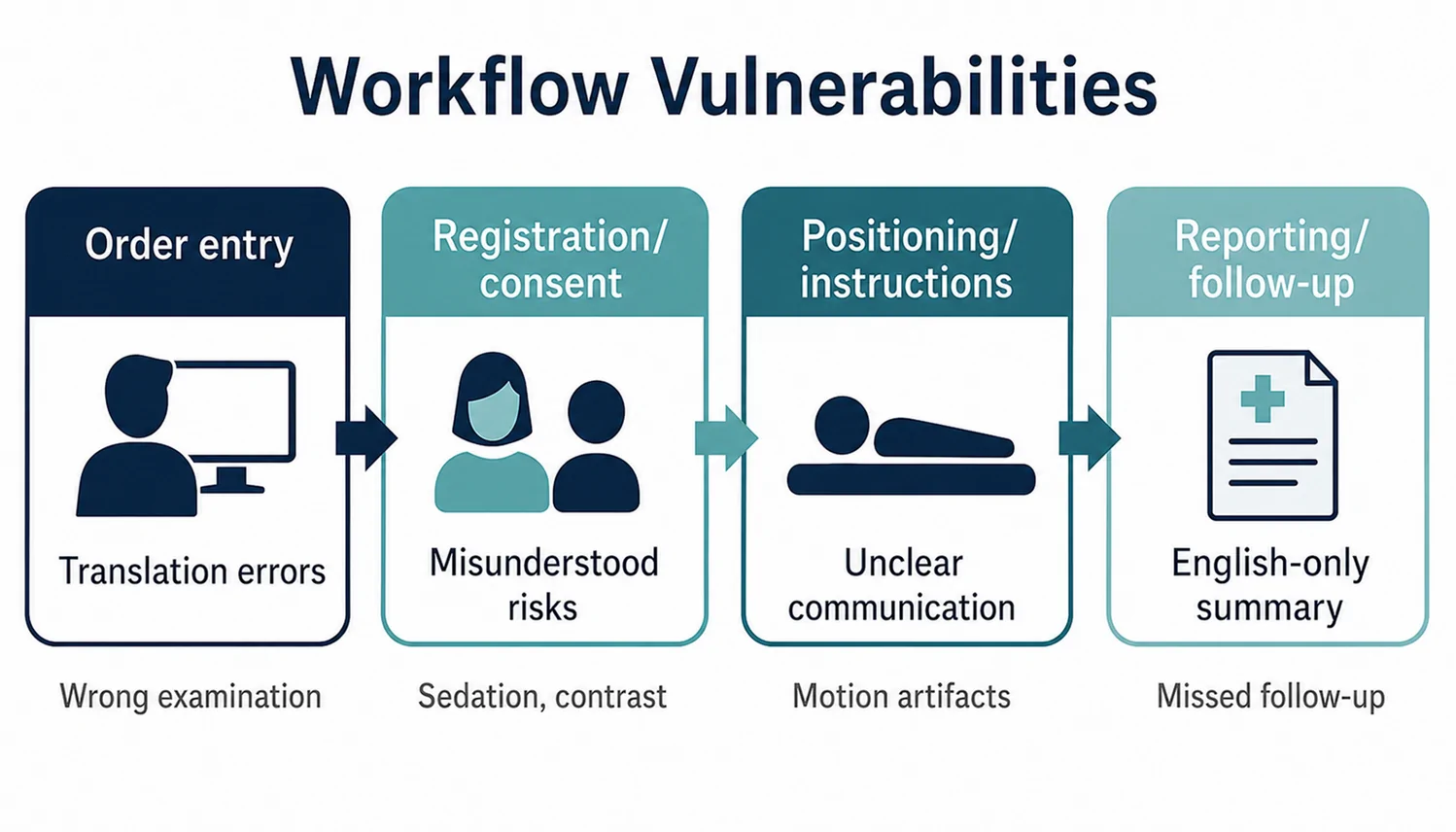
Language barrier failures mapped across the radiology care continuum — from order entry to reporting and follow-up. Each stage carries risks (translation errors, misunderstood consent, unclear instructions, English-only reporting) that can compromise patient safety, demonstrating the systemic need for multilingual and localized solutions

*Language* remains a major determinant of whether pediatric imaging AI becomes usable and safe. Most tools are designed and documented in English, yet Africa alone is home to approximately 1,500 to 3,000 languages [37]. In routine care, clinicians and families often communicate in local languages even when they received their medical training in English, which creates documented risks of miscommunication and reduced care quality when services are not delivered in the language of preference [38]. In pediatric settings, triadic communication (caregiver-child-clinician) amplifies these risks, particularly for safety-critical decisions around sedation, contrast administration, and radiation exposure (Fig. 2). Practically, Fig. 2 helps map where language-related failures may occur across the imaging pathway so that sites can target mitigation strategies at the highest-risk steps. Multilingual natural language processing models and datasets underrepresent many African and South Asian languages, with persistent accuracy gaps in health contexts [39]. Speech systems also show accent and dialect biases that can degrade dictation or voice-driven interfaces used in radiology workflows [40].

**Fig. 3:**
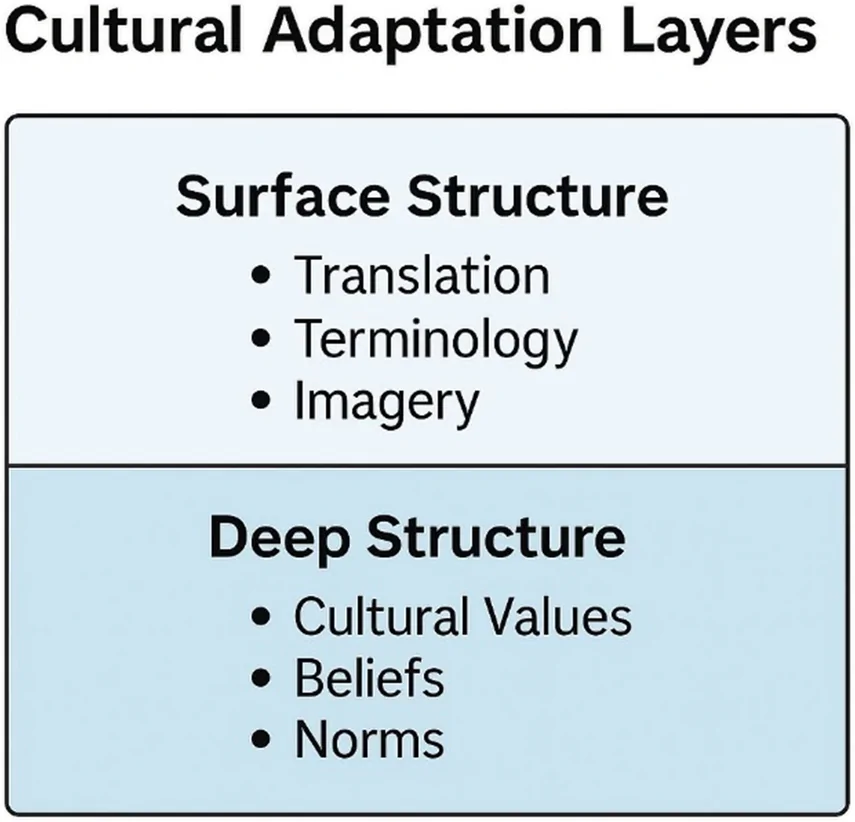
Two layers of cultural adaptation for AI in pediatric radiology: surface structure (translation, terminology, imagery) and deep structure (cultural values, beliefs, norms). Addressing both is essential for interventions to be not only linguistically accurate but also contextually trusted and ethically grounded

Localization is not only about words. *Cultural factors* such as parental decision-making norms, respect for authority, gender roles, and community perceptions of radiation risk shape how families interpret imaging recommendations and AI explanations. So, culturally neutral or English-centric outputs are prone to undermine trust and informed consent in LMIC pediatric settings [41]. Figure 3 provides a simple framework for applying localization in practice by distinguishing surface adaptations, such as translation and terminology, from deeper cultural adaptations that influence trust, consent, and decision-making (Fig. 3).

#### Solutions and enablers

Recent initiatives illustrate both progress and remaining gaps. India’s Bhashini mission is building a national infrastructure for speech recognition and translation across major Indian languages, enabling downstream health applications that can operate beyond English [42]. In East Africa, a Swahili large language model has been released to support maternal and child health messaging and could be adapted for radiology-adjacent tasks such as patient education and consent [43]. Within radiology, early studies show that large language models can translate and simplify reports across several languages, but variable performance for low-income languages and domain-specific terminology warrants careful validation before clinical use [44, 45].

**Fig. 4:**
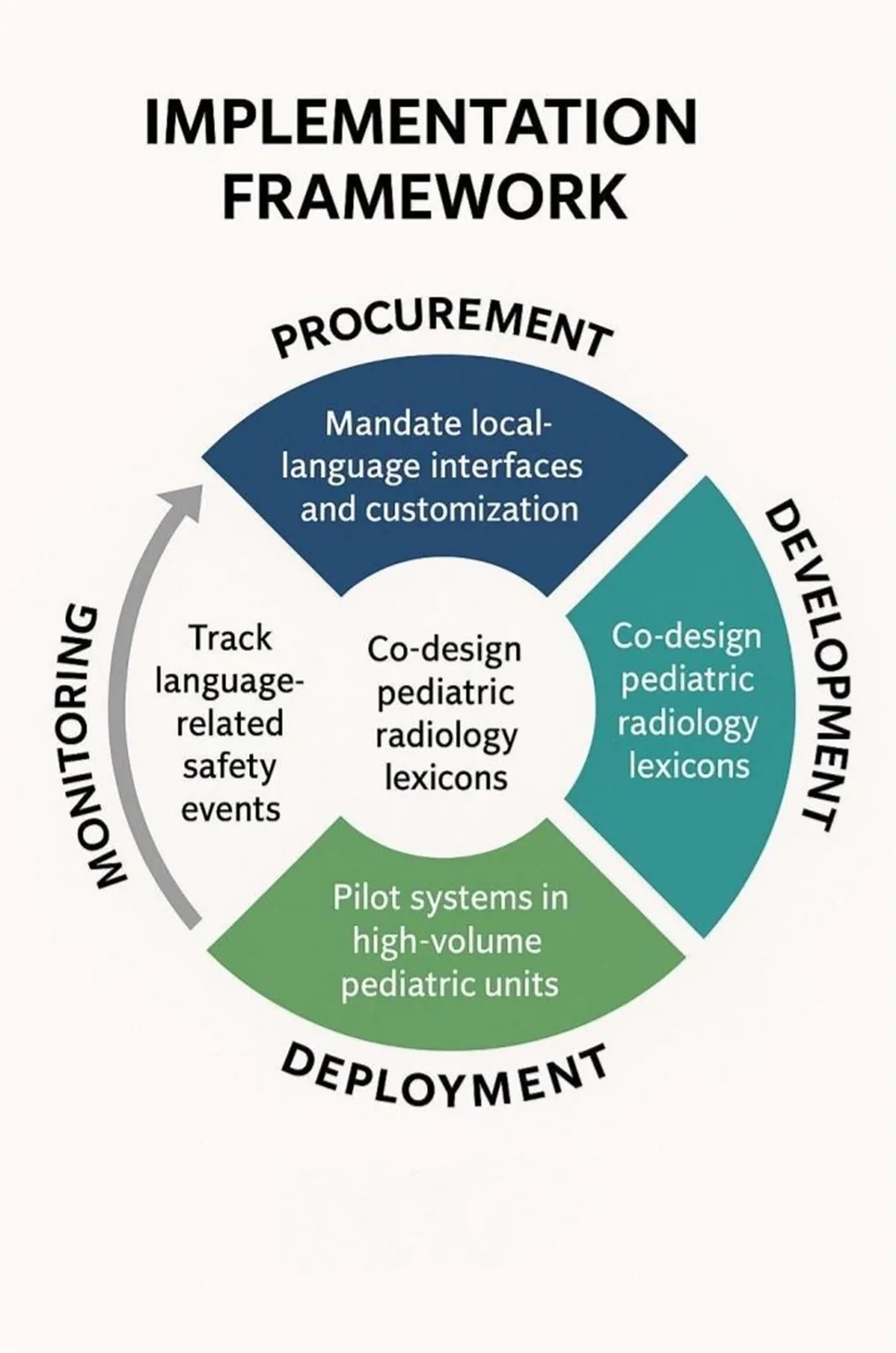
A cyclical framework for integrating pediatric AI into diverse settings: procurement (mandating local-language customization), development (co-designing pediatric lexicons), deployment (piloting in high-volume pediatric units), and monitoring (tracking language-related safety events). This pragmatic roadmap emphasizes iterative design and context-specific evaluation

A practical path forward includes multilingual user interfaces and outputs for clinician-facing and patient-facing modules, beginning with widely used regional languages (Fig. 4). Procurement should require local-language user interface (UI), instructions for use, and training materials. Developers should co-design pediatric radiology lexicons with local experts to preserve nuance and ensure consistency in report templates. National language programs such as Bhashini and regional natural language processing (NLP) communities can supply corpora and benchmarks for continuous model improvement [39, 42]. Systems should support robust speech and text input tolerant of accent and code-switching, with human oversight for safety-critical steps. Implementers should follow the WHO digital health guidance on cultural and linguistic adaptation, including adaptation kits, user testing with frontline workers, and monitoring for language-related errors after deployment [41, 46]. A practical strategy is to co-design pediatric imaging AI with local clinicians and caregivers and to use WHO SMART Digital Adaptation Kits to localize content and workflows, complemented by cultural-competency assessments like RAD-AID Radiology Readiness [15, 47]. Deployment should be done in phased pilots with iterative refinement based on user feedback.

### Costs and commercialization

Many AI tools are developed by commercial vendors and are expensive. High *licensing and maintenance costs* can prevent adoption in LMIC settings. Consequently, the wealthier healthcare systems stand to benefit disproportionately from these tools, while underserved regions risk lagging behind further. In LMIC settings, constraints extend beyond price to include infrastructure limitations, workforce capacity, and system instability, which can make lifecycle costs and sustained support even more difficult to absorb.

Beyond licensing, adoption of AI requires *costs for each of the steps in the AI lifecycle*, including pre-deployment evaluation, hardware and integration, governance personnel, and post-deployment safety monitoring [48, 49]. To the authors’ knowledge, a comprehensive analysis of costs across these steps has not been published to date. However, one report of deploying an AI platform for four tools estimated a 5-year cost of $1.7 million USD or $425,000 per tool, inclusive of labor, internal customer costs, and annual cost of service [50]. Notably, they reported a positive return on investment for the health system, based on additional procedures. For pediatric patients, one could expect the return on investment to be negative, given overall lower volume of imaging in pediatric patients [51].

A significant non-monetary cost is the *environmental impact* of AI. Specifically, powering AI, both during development and deployment in clinical use, utilizes substantial computational and energy resources, with projected negative sustainability effects [17, 52].

#### Solutions and enablers

While the monetary outlay of a single commercially available tool can be fairly straightforward to quantify, the total short-term costs are much more difficult to accurately tabulate but must be anticipated to be large. For institutions, a practical framework is to split costs into (a) fixed one-time up-front costs (i.e., interfaces, security reviews, contracting and ethical reviews, hardware/cloud connectors, etc.), (b) recurring fixed or variable costs (i.e., annual licenses or per-exam pricing, etc.), and (c) maintenance costs (i.e., hardware/cloud connector updates, IT troubleshooting, safety monitoring, etc.). Greater transparency of total cost of ownership and sustainable support models are important for equitable pediatric deployment.

### Sustainability and long-term support

*Long-term sustainability* of AI remains a pressing challenge in LMICs. AI systems demand continuous software updates, technical maintenance, and adaptation to evolving workflows, requirements that may exceed available infrastructure and expertise without local capacity and open-source accessibility [17, 53]. Many LMIC settings struggle to sustain these tools after initial deployment due to unreliable funding and limited in-house technical expertise. In many cases, vendors have no local presence, limiting timely support and updates. In pediatric radiology, this challenge is compounded by a smaller market size and weaker financial incentives for child-specific AI solutions, resulting in far fewer available products compared to adult imaging. Consequently, many deployments remain confined to short-term pilots, and even effective AI tools can become obsolete without planned updates, recurring funding, and local troubleshooting capacity.

#### Solutions and enablers

Sustainability depends on embedding AI into existing health systems and building local capacity to maintain and adapt these tools. Key enablers include defined local responsibility in the hands of (pediatric) radiologists and technology staff, aligning deployment with clinical needs, and leveraging open-source or affordable licensing models. Building a local technical workforce, including training in-country IT specialists and radiologists in AI software management, enables sites to independently maintain and adapt AI systems. This includes continuous performance monitoring as this is equally critical to maintain clinical relevance and avert algorithmic drift [54]. These approaches are underscored in the ACR’s pediatric AI and ESPR AI Taskforce directives, as well as the updated Checklist for Artificial Intelligence in Medical Imaging (CLAIM) [9, 10, 55].

Anchoring deployment within global professional networks can also support sustainability through shared practices and ongoing mentorship. By sharing projects in professional networks, LMICs can reduce isolation, share best practices, and access ongoing mentorship and advocacy, ultimately supporting the long-term viability of AI-enabled imaging solutions. Finally, securing long-term financing models (such as public-private partnerships or integration into government budgets) is essential so that AI tools persist beyond just the pilot phase.

## Roadmap for equity-focused implementation

Table 2 summarizes the above discussed domain-specific actions and responsibilities. This roadmap section prioritizes a small set of high-impact steps in time order and distinguishes actions that are feasible within LMICs from actions that HICs can take to enable equitable progress.

**Table 2:** Domain action map for equity focused Artificial Intelligence (AI) implementation in pediatric radiology

Domain	Key barrier	Practical fix	Primary responsibility
1. Data inequality and bias in model development	Pediatric and LMIC underrepresentation, subgroup performance gaps	Build diverse pediatric datasets, require stratified reporting and external validation, use federated learning where appropriate	Developers and vendors; hospitals and health systems; professional societies; funders
2. Infrastructure disparities	Unreliable power, limited digitization, weak connectivity, limited compute	Phased digital readiness upgrades, offline or edge workflows, standards-based interoperability	Hospitals and health systems; governments and regulators; vendors; funders
3. Regulatory and ethical gaps	Unclear governance for data sharing, consent, cloud use, and accountability	Adopt data governance and sovereignty frameworks, define cloud and on premises rules, mandate monitoring and accountability	Regulators and governments; hospitals and health systems; professional societies; vendors
4. Workforce capacity and training gaps	Shortage of pediatric expertise and limited AI governance skills	Embed AI competencies in training, create protected clinical AI roles, enable multidisciplinary teams	Hospitals and health systems; professional societies; training bodies; funders
5. Language and localization barriers	English centric tools, low resource language gaps, cultural mismatch	Local language UI and documentation, validated lexicons, human oversight for safety critical communication	Vendors and developers; hospitals and health systems; professional societies; governments
6. Costs and commercialization	High licensing costs, hidden lifecycle costs, limited LMIC affordability	Transparent total cost of ownership, tiered pricing and public procurement models, open and modular options	Vendors; hospitals and health systems; funders; governments and regulators
7. Sustainability and long term support	Weak maintenance capacity, vendor dependence, limited post deployment monitoring	Local capacity building for maintenance, service level agreements, continuous drift and equity monitoring	Hospitals and health systems; vendors; professional societies; funders

*AI*, artificial intelligence, *LMIC*, low- and middle-income countries, *UI*, user interface

In the near-term, the priority is safe and realistic adoption. In LMICs, this means selecting a small number of locally relevant use cases that can function with limited connectivity, establishing basic local governance for intended use and incident review, and ensuring local language interfaces and training materials for clinician and caregiver communication. In HICs, the most important enabling actions are to normalize pediatric-specific procurement and publication standards that require stratified performance reporting and external validation, and to support equitable partnerships that provide technical assistance and shared labeling standards.

In the mid-term, the priority is capacity building and scalable validation. In LMICs, this means phased upgrades in digitization and interoperability, development of multidisciplinary teams with protected clinical leadership for AI oversight, and participation in multicenter validation or federated learning networks that respect data sovereignty. In HICs, this means sustained investment in representative pediatric datasets across age bands and regions, harmonized expectations for pediatric claims and monitoring, and cost models that improve affordability and reduce vendor dependency.

In the long-term, the priority is sustainability and continuous safety. In LMICs, this means local maintenance capacity, service agreements, and routine monitoring for drift and subgroup performance with clear pathways for updates. In HICs, this means shared governance frameworks, long-term financing mechanisms, and global benchmarking that includes equity endpoints and real-world deployment constraints.

Trackable indicators include stratified performance reporting by pediatric subgroups, external validation across regions, infrastructure readiness and uptime plus latency targets, completion of role-appropriate training and presence of a local clinical AI lead, post-deployment drift monitoring with documented update or rollback procedures, language and localization error reporting, and documented total cost of ownership with a sustainability plan beyond pilot funding.

## Conclusions

Over the past decades, AI innovation and deployment in medical imaging have risen dramatically, yet its benefits are not equitably distributed across the globe. Achieving global equity in pediatric AI requires coordinated action across seven domains, namely data inequality and bias, infrastructure disparities, regulatory and ethical governance, workforce and training capacity, language and localization barriers, costs and commercialization, and sustainability and long-term support. Key priorities include developing diverse pediatric datasets and reporting performance in a stratified manner, with external validation across different regions. They also include ensuring context-appropriate infrastructure and governance, investing in multidisciplinary training, and planning for affordable long-term maintenance and monitoring. By synthesizing these actions and responsibilities and outlining time-sequenced priorities, this paper provides a practical, equity-focused framework to support safe global implementation of pediatric imaging AI.

## Data Availability

No datasets were generated or analysed during the current study.
